# AI-integrated bionic fingertip E-Skin for precision slippage detection in wet environments

**DOI:** 10.1038/s41598-026-41096-z

**Published:** 2026-03-19

**Authors:** Tsubasa Adachi, Koki Ozawa, Shoma Kamanoi, Junya Yoshida, Riku Sasaki, Yasuyuki Miura, Yoshihito Takabe, Fabrice Domingues Dos Santos, Tong Huang, Atsushi Miyabo, Yasunori Takeda, Hiroyuki Matsui, Tomohito Sekine

**Affiliations:** 1 Graduate School of Organic Materials Science, Yamagata University, 4-3-16, Jonan, Yonezawa, 992-8510 Yamagata Japan; 2 Arkema Piezotech, Pierre-Benite Cedex, F-63493 France; 3 Arkema K. K, 2-2-2 Uchisaiwaicho, Chiyoda-ku, Tokyo, 100-0011 Japan; 4 Innovation Center for Organic Electronics (INOEL), Yamagata University, 1-808-48 Arcadia, Yonezawa, 992-0119 Yamagata Japan

**Keywords:** Engineering, Materials science, Optics and photonics, Physics

## Abstract

Electronic artificial skin (E-skin) replicates human tactile sensations with exceptional sensitivity and accuracy, enabling the detection of physical properties, including the shape, material, and texture of objects. Current technologies effectively detect slippage on dry surfaces but not on oil- or water-coated wet surfaces. This paper presents a wearable slip sensor featuring a micropatterned structure inspired by human fingerprints, capable of detecting slippage under all surface wetness conditions. The proposed sensor incorporates a randomly patterned fingerprint design, laser-etched onto the topmost layer of a multilayer film. It effectively detects surface slippage, even on oil film-coated low-friction surfaces. Additionally, the sensor captures intricate geometric features of microtextures, including microvibrations and ultrafast signal changes. Its applicability in soft robotic hands is demonstrated by its high-speed detection of the sliding motion of various objects. The findings will aid in advancing digital-on-demand technologies by enabling the precise reconstruction of digital tactile data within cyber-physical systems.

**Supplementary Information:**

The online version contains supplementary material available at 10.1038/s41598-026-41096-z.

## Introduction

Human skin is a densely packed network of multifunctional sensors capable of responding sensitively to external physical stimuli. Researchers have long focused on developing high-performance electronic artificial skins (E-skins) that replicate the sensing capabilities of human skin. Modern E-skins can now detect a wide range of physical stimuli, including acceleration^1,2^ and tensile stress,^3,4^ in addition to fundamental tactile sensations such as pressure,^5–8^ temperature,^9–12^ and humidity.^13–16^ They also hold significant potential for various applications, including prosthetic hands interfacing with neural systems^19,20^ and human–machine interfaces.^21,22^ Despite these advancements, several challenges remain in achieving a tactile perception system as precise as human skin. Key obstacles include the automatic processing of signals using artificial intelligence (AI) and the multimodal integration of sensor devices.^13,23,24^ In particular, detecting object slippage is a major challenge and is essential for maintaining a secure yet non-damaging grasp while preserving the object’s shape. Tactile sensors function by converting mechanical or physical stimuli—such as pressure, bending, or temperature changes—into electrical signals. Various types of pressure sensors have been developed, including piezoresistive,^25,26^ capacitive,^27,28^ and piezoelectric^29,30^ sensors. Additionally, other sensor technologies, such as frictional electric^31^ and optical^32^ sensors, have been explored. Recently, an array-type slip sensor^33^ was introduced for robotic applications, representing a crucial advancement in the accurate detection of an object’s texture during grasping. Slip sensors are primarily of two types: static slip sensors, which detect stress changes at the onset of slippage, and dynamic slip sensors, which capture minute vibrations at the interface between the object and the sensor during slippage. Static slip sensors are advantageous for their array configuration and precise slip detection, while dynamic slip sensors enable real-time monitoring of slip phenomena during sliding. Both technologies are essential for enabling robots to grasp objects accurately. However, dynamic slip sensors, in particular, play a critical role in force control technology, allowing robots to manipulate objects with precision. Advancing AI-driven recognition mechanisms is fundamental to robotic sensing technology. The integration of AI with tactile sensors is essential for enhancing the functional capabilities of robotic tactile perception, highlighting the need for continued collaboration between sensor technology and AI.^34^

Dynamic slip sensors have been developed primarily for robotic hand applications. One example is a highly sensitive wearable sensor created by softening ferroelectric material into a sponge-like structure.^35^ Another example involves a dual optical and electrical sensor designed to detect dynamic slip phenomena with high accuracy in a wearable robotic setup.^36^ These sensors exhibit exceptional sensitivity and rapid detection speeds. However, their application has so far been limited to detecting objects in dry conditions. This limitation poses a challenge for robotic systems that must handle a wide range of objects in diverse environments. For instance, in culinary tasks, advanced slip sensor technology is crucial for securely grasping objects that are coated with oil or other slippery substances. To achieve human-level dexterity, robots must accurately detect slippage even when an object’s surface is covered with a water or oil film. Attaining this capability is essential for enabling precise and adaptable robotic grasping in real-world scenarios.

Furthermore, the fabrication of flexible and wearable sensors holds significant engineering value. Various process engineering studies have explored sensor fabrication using two-dimensional (2D) and three-dimensional (3D) printing techniques.^37^ In this study, screen printing was selected owing to its high printing efficiency, material flexibility, suitability for mass production, and cost-effectiveness. Furthermore, this method is an on-demand, resource-efficient, and energy-saving approach, addressing the urgent environmental concerns in the electronics industry. Printed electronic device fabrication offers a promising pathway toward achieving sustainability goals, such as those outlined in the sustainable development goals.^38^ The fully printed sensor devices presented herein demonstrate the potential for highly integrated printed electronics in future applications, including human-robot interfaces, intelligent robotics, and E-skins.

This study developed a wearable slip sensor featuring a micropatterned structure inspired by human fingerprints, designed to detect slip phenomena under any surface wetness condition. The proposed device incorporates a laser-processed random fingerprint pattern on the topmost layer of a multilayer sensor, enabling accurate detection of sliding motions by simply tracing the surface of an oil-coated film, even under low-friction conditions. This structural design enhances the detection of dynamic slip even in wet environments, as the exclusion effect of the liquid increases friction at the interface between the sensor and the object.

Additionally, our tactile sensor exhibits high sensitivity in detecting dynamic slip owing to its ability to capture microvibrations generated by the piezoelectric response of ferroelectric polymers. We successfully obtained comprehensive geometrical information on microtextures, including ultrafast signal detection related to slippage, and applied this capability to E-skin for robotic hands. Furthermore, the slip-related signals were converted into big data and analyzed using machine learning, enabling a high level of object identification. These findings highlight the potential of fingerprint-inspired micropattern tactile sensors in shaping a future where quantified digital tactile data are reconstructed within cyber-physical systems, contributing to the vision of a digital-on-demand world.

## Results and discussion

**Fig. 1:**
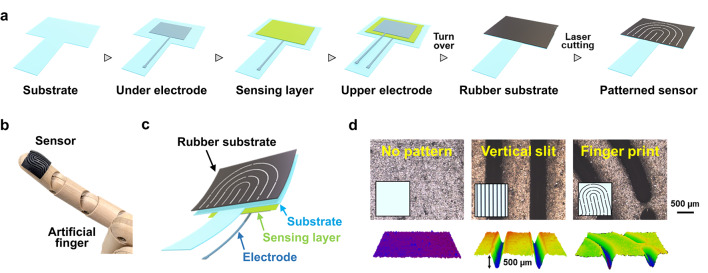
Highly sensitive wearable slip sensor inspired by fingerprints. (**a**) Sensor fabrication process using an all-screen-printing method. (**b**) Top-down view of the fabricated sensor. (**c**) Cross-sectional view of the sensor. The fingerprint pattern was created by mounting a rubber substrate on a capacitor-type sliding sensor and engraving it with a laser cutter. (**d**) Surface morphology and cross-sectional profile of the rubber substrate with fingerprint pattern. The pattern grooves are approximately 400 and 500 μm in height and width, respectively. The insets in the photographs show images of the respective patterns.

The developed sensors are designed for wearable devices and can be seamlessly integrated into both human and robotic systems owing to their soft and flexible nature. Figure 1 provides an overview of the sensor design and fabrication process. As depicted in Fig. 1a, the sensors are fabricated using an all-printing-based method, emphasizing sustainability and ubiquity in sensor technology. The basic structure consists of a capacitor with a ferroelectric polymer layer sandwiched between electrodes. Additionally, slip-detection functionality is enhanced by imprinting fingerprint-like micropatterns onto the surface using a CO₂ laser cutter.

A small amount of nanocarbon material, specifically single-walled carbon nanotubes (SWCNTs), was incorporated into the ferroelectric material to enhance the sensor’s sensitivity to sliding phenomena.^39^ The detailed fabrication process of the sensor is shown in Fig. 1b. A polyethylene naphthalate (PEN) film (Teonex Q8300, Teijin) was used as the substrate, with a thickness of 50 μm. The conductive polymer PEDOT: PSS (Clevios SV4 STAB, Heraeus) was first deposited onto the PEN film via screen printing and subsequently annealed at 150 °C for 30 min, forming an electrode layer with a thickness of 500 nm. Next, a solution of P(VDF-TrFE) (FC25, Arkema-Piezotech) was prepared in N-methyl-2-pyrrolidone at a concentration of 12 wt%, with SWCNTs mixed at 0.075 wt% relative to P(VDF-TrFE). This solution was deposited onto the bottom electrode via screen printing and annealed at 140 °C for 1 h, forming a dielectric layer with a thickness of 5000 nm. The top electrode was fabricated using the same method as the bottom electrode and annealed at 140 °C for 30 min. To provide protection, the same material as the base layer was spin-coated onto the top electrode, maintaining a consistent thickness. A butyl rubber substrate (1 mm thick) was attached to the back of the fabricated sensor, and its surface was micropatterned using a CO₂ laser processing machine (Beamo, Maverick) with an output power of 25 W. The fingerprint-like pattern was designed as a random structure mimicking human fingerprints, along with a simple straight-line pattern. The fabricated sensor, shown in Fig. 1b, is highly flexible, allowing easy integration into artificial fingers or robotic systems. Additionally, as the device was produced using a printing-based fabrication method, it offers high design flexibility to accommodate the size and shape of the artificial fingers onto which it is mounted. The cross-sectional structure of the sensor is depicted in Fig. 1c. The grooves of the fingerprint pattern measure approximately 400 μm in width and 500 μm in depth. The relationship between laser power and pattern dimensions is presented in Supplementary Figure S1, while Fig. 1d depicts the surface morphology and cross-sectional profile of the fabricated pattern. The engraving conditions were optimized to achieve the cleanest pattern formation for a given laser output (Supplementary Figure S2). As a ferroelectric device, the sensor’s slip detection sensitivity was further enhanced via poling treatment, conducted at 1 Hz under an electric field of ± 5 kV. This process enables the sensor to detect microvibrations generated at the sensor-object interface, which are converted into voltage signals upon the occurrence of slippage. Because the device exhibits piezoelectric behavior, a poling process is required prior to use. Once this poling treatment is performed, no external power supply is needed during subsequent operation.

**Fig. 2:**
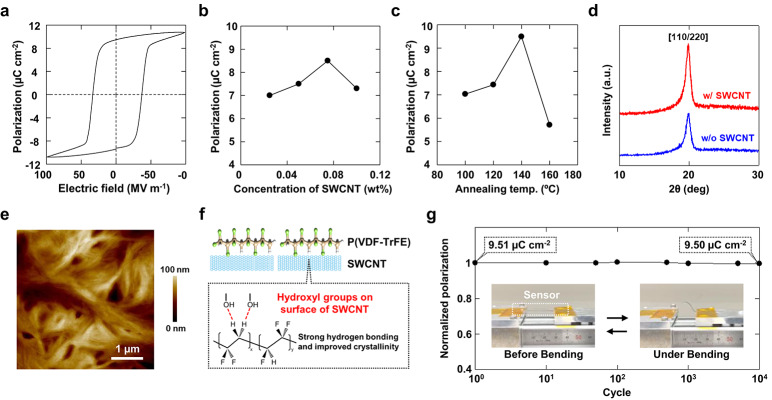
Basic characteristics of the fabricated sensor. (**a**) Hysteresis curve of the sensor, where the Pr value is the value of Y axis at 0 MV/m. The Pr of the pure sensor was approximately 7 µC/cm^2^: however, the addition of SWCNTs improved it to approximately 9.5 µC/cm^2^. (**b**) Variation of Pr value with SWCNT content; Pr value increased with increasing SWCNT content but decreased above 0.1 wt%. (**c**) Relationship between device annealing temperature and Pr value, with the highest performance observed at 140 °C, influenced by the crystallization temperature of the sensing layer. (**d**) X-ray diffraction spectrum of the sensing layer; the crystallinity was improved by adding SWCNTs to P(VDF-TrFE). (**e**) AFM image of the SWCNT-containing sensing layer surface. The RMS surface roughness was 35.4 nm. (**f**) Schematic image of chemical bonding at the micro interface between P(VDF-TrFE) and SWCNTs. Hydrogen bonding at the interface of each material improves the crystallinity of P(VDF-TrFE), resulting in improved ferroelectric properties. (**g**) Change in electrical properties under repeated mechanical stress. The device was subjected to a continuous compressive strain of approximately 1.5% by bending for up to 104 cycles. The inset photographs represent the actual bending test. Furthermore, the simulation figures visualize the stress distribution within the device at that time. The units for the color bars in both cases are 10³ N/m².

The developed wearable slip sensor is a high-performance device capable of detecting microvibrations generated by ferroelectricity. When slippage occurs at the interface between the object and the sensor, minute vibrations are produced, which can be detected as voltage signals. Because these microvibration signals are inherently weak, we first enhanced the sensor’s performance by increasing its ferroelectricity. Figure 2 shows the basic characteristics of the fabricated device. Figure 2a displays the hysteresis curve representing its ferroelectric properties. The residual polarization (Pr) is herein defined as the polarization value at an electric field of 0 MV/m. The Pr value of the undoped sensor was approximately 7.5 µC/cm^2^, whereas the sensor incorporating SWCNTs exhibited an increased Pr value of approximately 9.5 µC/cm^2^. This enhancement in Pr indicates improved sensitivity to vibration, demonstrating that this material design is highly effective for detecting slip phenomena.^40^ Notably, achieving a Pr value of nearly 10 µC/cm^2^ through a printing-based fabrication method is exceptionally rare, highlighting the potential for fabricating ultra-high-performance wearable devices.^41^ Fig. 2b illustrates the relationship between SWCNT content and Pr value. As SWCNT concentration increased, the Pr value also increased but declined beyond 0.1 wt%. This decrease is attributed to the increased conductivity within the P(VDF-TrFE) layer, which reduces the material’s insulation and dielectric properties as a capacitor.^42^ Fig. 2c shows the effect of annealing temperature on Pr value, with the highest performance observed at 140 °C. This optimal temperature corresponds to the crystallization temperature of the sensing layer. Beyond 140 °C, the β-phase of P(VDF-TrFE) began to degrade, leading to a significant reduction in dielectric properties.^43^ Fig. 2d shows the X-ray diffraction analysis of the sensing layer, confirming that the incorporation of SWCNTs enhanced the crystallinity of P(VDF-TrFE). This improvement is likely due to interactions between the functional groups of P(VDF-TrFE) and SWCNTs, which promote an increase in β-phase crystallinity.^44,45^.

Figure 2e presents an atomic force microscopy (AFM) image depicting the surface of a SWCNT-enriched ferroelectric layer, with the root mean square (RMS) surface roughness measuring 35.4 nm. This relatively flat surface enables the multilayer device to maintain excellent electrical properties despite the generally lower yield of the printing method. Figure 2f further displays the chemical bonds at the micro-interface between P(VDF-TrFE) and SWCNTs, where hydrogen bonding enhanced the crystallinity of P(VDF-TrFE), thereby improving its ferroelectric properties.^39^ Fig. 2g examines the electrical properties under repetitive mechanical stress, showing that the Pr values remained stable, even with a continuous application of approximately 1.5% compressive strain by bending over 10^4^ cycles, demonstrating the sensor’s exceptional mechanical durability. Moreover, using the finite element method (COMSOL Multiphysics^®^), we constructed a simulation setup identical to the bending experiment and simulated the strain distribution (inset Figures in Fig. 2g). The results revealed that bending leads to a uniform distribution of strain throughout the device, indicating that the absence of local stress concentrations is one of the factors contributing to the long mechanical lifespan of the device.

**Fig. 3:**
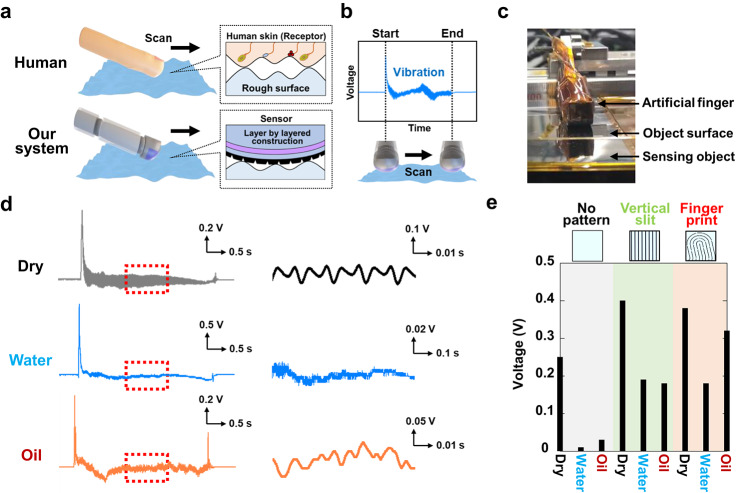
Slip test using a high-sensitivity sensor. (**a**) Schematic of material/texture recognition for a robotic prosthesis. (**b**) Dynamic slip signal generated when the sensor was mounted. Periodic voltage signals occurred during the scanning of the artificial finger. (**c**) Photograph of a setup where a sensor with a fingerprint pattern was mounted on an artificial finger. (**d**) Real-time detection of the slip signal using a stainless-steel plate as the sensing object under three conditions: dry, water-wetted, and oil-wetted. The left panel displays the signal captured by scanning the artificial finger from the start to the end of the slip, showing periodic voltage waveforms due to the S-S phenomenon. The enlarged section marked by the red dotted lines is displayed in the right panel. It clearly demonstrates periodicity, with distinct differences observed in the periodicity for dry, water-wetted, and oil-wetted conditions. (**e**) Signal intensities from fingerprint-patterned sensors under dry, water, and oil conditions exhibit significantly enhanced sensitivity compared to those from non-patterned sensors. Notably, the fingerprint pattern enables precise signal detection even in low-friction environments such as those in oil conditions.

Figure 3 demonstrates the sensor mounted on an artificial finger for detecting slippage on a stainless-steel plate during a slip test. Figure 3a shows the slip test in progress. The human fingertip, which contains numerous tactile receptor cells for sensing mechanical stimuli, inspired the design of the sensor. The vibration-sensing function of these biological receptors was emulated using an electronic device that incorporated a fingerprint pattern on the topmost sensor layer. The test object surfaces were subjected to three different conditions: dry, water-wet, and oil-wet. The device was aligned such that the patterned layer made direct contact with the test surface, operating at a longitudinal speed of 50 mm s⁻¹ while applying a constant vertical force of approximately 1 N. The sensor’s responses to varying scanning speeds are detailed in Supplementary Figure S3.

Under these experimental conditions, the effectiveness of the fingerprint pattern on the topmost surface in enhancing slip signal detection was evaluated. Figure 3b displays the voltage signal captured during slippage, which exhibited a periodic waveform from the onset to the end of the slip event. The signal amplitude was influenced by both the sensor’s sensitivity and the surface roughness frequency of the test object. Figure 3c depicts the sensor affixed to an artificial finger in contact with a stainless-steel plate. This setup enabled real-time slip detection under dry, water-wet, and oil-wet conditions, as shown in Fig. 3d. The left graph in Fig. 3d shows the recorded voltage signal as the artificial finger moved across the surface. In dry conditions, a pronounced signal exceeding 0.1 V was observed, reflecting the sensor’s high sensitivity to microvibrations during slippage. Notably, comparable signal amplitudes were also recorded under water and oil immersion. The right graph provides a magnified view of the region indicated by the red dotted box, where periodic signals are visible in all three conditions. This periodicity originates from the stick-slip (S-S) phenomenon at the interface between the sensor and the object,^46^ confirming that the sensor can detect clear slip signals even in low-friction environments such as those presented by water and oil. These signals exhibit sinusoidal waveforms with intervals of approximately 0.1 s. Fourier transformation revealed that they correspond to fine, high-frequency components in the range of 100 Hz. The sensor demonstrated an exceptional ability to capture these delicate signals accurately, even under wet conditions. The results of the fast Fourier transform (FFT) performed to analyze the frequency components of the slip signal in Fig. 3d are presented in Supplementary Figure S4. The peak frequencies for all conditions were approximately 100 Hz. This suggests that the frequency associated with the S–S phenomenon generated during slipping is independent of the wetness condition of the objects. Figure 3e depicts the comparative advantages of fingerprint-patterned sensors under dry, water, and oil conditions. Unlike the unpatterned sensor, which exhibited limitations in detecting signal amplitudes under wet conditions, the patterned sensor maintained effective performance. Fingerprint-patterned sensors effectively detect signals even in low-friction environments, such as those in oil conditions. Furthermore, the fingerprint pattern enhanced signal amplitude in low-viscosity environments, such as oil, due to the drainage effects of grooves strategically aligned with the direction of finger movement.^47^ In conclusion, the deployment of a highly sensitive slip sensor incorporating a fingerprint pattern enabled precise detection of slippage across a variety of surface conditions.

**Fig. 4:**
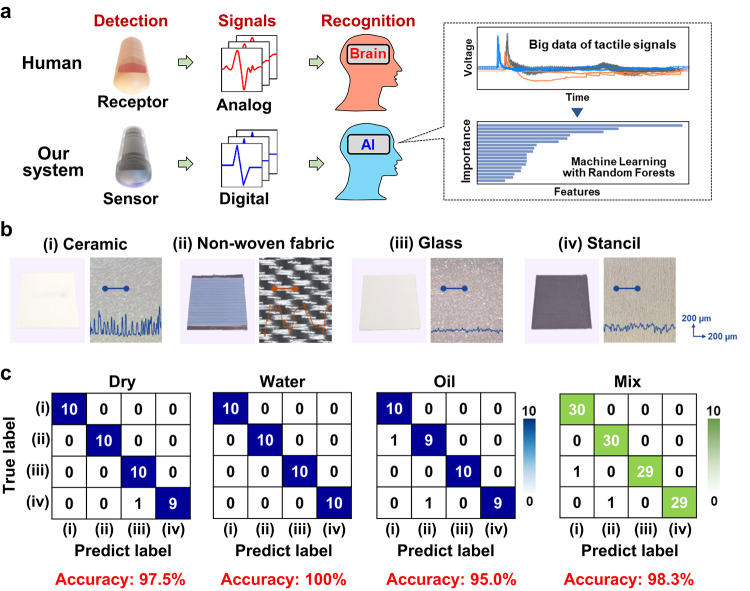
Object classification based on features of slip signals using a machine learning algorithm. (**a**) Diagram illustrating the recognition mechanism of humans and AI equipped with sensors, grounded on sliding phenomena over object surfaces. (**b**) Photograph of the object and its surface morphology. The blue line area in the photograph is profiled as an optical microscope image on the right. (**c**) Error table for automatic classification of slip signals obtained through machine learning. Fingerprint-patterned sensors were utilized to classify signals from objects under dry, water-wetted, and oil-wetted conditions, achieving over 95% classification accuracy in each scenario.

Machine learning was employed to automatically identify surface materials. Figure 4a depicts a schematic diagram of the AI-based automatic identification process. In the human somatosensory system, signals from mechanoreceptors in the fingertips are transmitted as continuous analogue information and processed in the brain as graded spatiotemporal patterns^48^,^49^. The proposed machine learning model demonstrates the effectiveness of digitally processed signals from the sensor in material identification systems. For this study, the range of test materials was expanded to include, in addition to stainless steel, ceramic, non-woven fabric, and glass plates. Figure 4b presents the surface morphology of these materials, with cross-sectional profiles of selected areas shown as insets. Ten scans were performed for each object under each condition (dry, water-wet, and oil-wet) using two fingerprint-patterned sensor devices, yielding a total of 120 signal waveforms. Representative waveforms for each object and condition are shown in Supplementary Figure S5. A machine learning model based on the random forest algorithm was developed to classify surface materials by aggregating decisions from multiple decision trees. The model was implemented in Python using the scikit-learn library. Eighteen features were extracted from the raw sensor waveform and its Fourier transform, including the mean, median, first quartile, third quartile, standard deviation, minimum, maximum, skewness, kurtosis, and the frequency at which the Fourier amplitude was maximized (Supplementary Figures S6 and S7. The model’s classification performance was evaluated using 5-fold cross-validation. Figure 4c shows the results, with the dataset separated by wetness condition prior to model training. We utilized the class-wise stratified 5-fold cross-validation (StratifiedKFold). This method randomly partitions the dataset into five folds while maintaining the same class distribution in each fold, enabling balanced and reliable model evaluation. The results show that the sensor consistently achieved high classification accuracy, exceeding 95% across all surface conditions. This performance was attributed to the sensor’s ability to generate distinct signal waveforms, which varied in amplitude according to wetness and in frequency according to the surface material. Furthermore, in a comprehensive test involving all 120 mixed-condition signals (Far-right panel of Fig. 4), the sensor achieved an overall classification accuracy of 98.3%. This result highlights both the high-resolution capability of the sensor in detecting slippage and the effectiveness of the proposed machine learning algorithm in accurately classifying signal features. A subsequent analysis of feature importance revealed that the frequency corresponding to the maximum Fourier transform, the third quartile of the raw waveform, the kurtosis of the Fourier transform, and the maximum value of the Fourier transform were the most influential features in automatic classification.

**Fig. 5:**
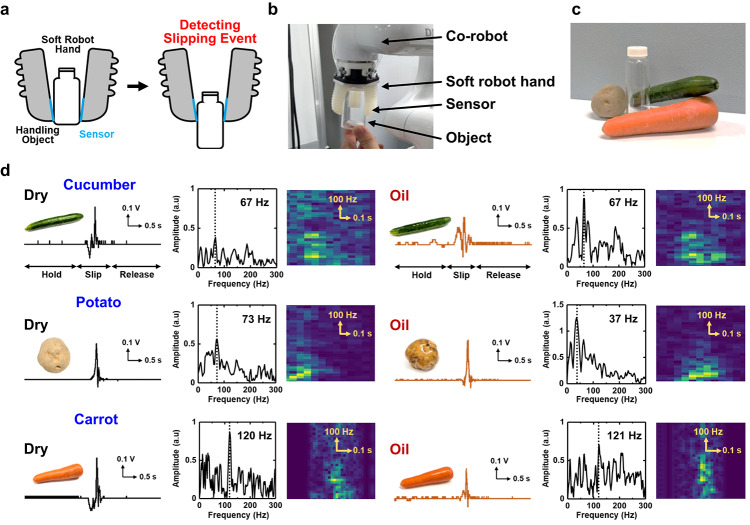
Soft robot hand application of a slip sensor as wearable E-skin. (**a**) Schematic images of slip detection using a soft robotic hand. (**b**) Photograph of an actual object being grasped by the soft robotic hand with the sensor mounted. (**c**) Photograph of the actual objects used in this demonstration: cucumber, potato, and carrot. (**d**) Real-time signals corresponding to the slippage of each object detected by the sensor-equipped soft robotic hand. All signals exhibited characteristic periodic waveforms, which are shown on the left side of each object’s data group. The Fourier spectra obtained by applying the FFT to the slip segments of each signal are presented in the center of each data group, with the frequency exhibiting the highest amplitude inset in each FFT plot. The FFT analysis was conducted on an arbitrary 0.75 s window of the slip signals. The FFT analysis was conducted on the slip signal after differentiation. Furthermore, the STFT of the slip segments is shown on the right side of each object’s data group. The inset photos show the objects under dry and oil-wetted conditions, respectively.

Figure 5 illustrates the application of the sensor in an E-skin configuration on a soft robotic hand. A sensor featuring fingerprint-like patterns was attached to the surface of a polydimethylsiloxane-based soft robot, enabling real-time detection of vertical slippage as the robot grasped various objects. Figure 5a shows the slip detection process using the soft robotic hand, which was designed and fabricated with a 3D printer based on previous research.^47^ Fig. 5b presents the soft robotic hand grasping arbitrary objects with the sensor mounted. These objects were tested under both dry and oil-wetted conditions, and slip signals were recorded for each case. Figure 5c displays the actual objects used in the experiments, specifically cucumbers, potatoes, and carrots. Figure 5d shows the measured signals obtained from each object along with the corresponding frequency analyses. Real-time slip signals were successfully detected for all tested objects. Furthermore, all signals exhibited characteristic periodic waveforms, and applying the FFT to the slip segments enabled analysis of the signal periodicity associated with slippage events. Although acquiring slip signals from wet objects is generally challenging, this study successfully extracted object-dependent frequency components under both dry and oil-wetted conditions. In addition, Short-Time Fourier Transform (STFT) analysis revealed a clear correlation between the real-time signals and their time–frequency representations. These results demonstrate that the versatile E-skin is highly effective for soft robotic hand applications, functioning as a sensory organ with tactile perception capabilities comparable to, or even exceeding, those of human skin.^50^,^51^.

## Conclusions

This study introduced a slip sensor featuring a micropattern that mimics human fingerprints, designed to enhance tactile sensing capabilities. The sensor combines the basic properties of tactile sensors with practical applications, demonstrating operational speed and precision comparable to those of human tactile perception. This performance is attributed to the effective integration of organic materials with the high-precision electrical properties of the newly developed printed slip sensor. The sensor unit exhibited high sensitivity in capturing microvibrations (100 Hz) at the interface between the sensor and an object, achieving ferroelectric properties of approximately 9.5 µC/cm². The top surface of the sensor featured a laser-engraved random micropattern that closely resembled a human fingerprint, enabling the accurate detection of minute vibration amplitudes associated with dynamic slip events. The sensor demonstrated precise detection capabilities for objects coated with either water or oil. The output signals were efficiently processed using proprietary machine learning algorithms, which accurately classified objects based on surface slips. When integrated into a soft robotic hand, the sensor-enabled high-speed detection of sliding motions with excellent temporal resolution. The fabrication process employed a screen-printing technique, which facilitated the production of multilayered, wearable devices capable of detecting dynamic slip with high precision. Overall, the proposed slip sensor and its fabrication method provided a unique and promising outlook for advancing digital tactile sensing and contributed to the development of digital-on-demand environments that transformed quantified haptic data into tangible cyber–physical interactions.

## Supplementary Information

Below is the link to the electronic supplementary material.

Supplementary Material 1

## Data Availability

The data supporting this article have been included as part of the Supplementary Information.
