# Supplementary material for: AI-integrated bionic fingertip E-Skin for precision slippage detection in wet environments
Source: Sci Rep. 2026 Mar 19;16:14179. doi: 10.1038/s41598-026-41096-z (PMC13139447; doi:10.1038/s41598-026-41096-z)
Supplement: Supplementary file 1 — Supplementary Material 1 [file 41598_2026_41096_MOESM1_ESM.pdf]

AI-Integrated Bionic Fingertip E-Skin for Precision Slippage Detection in Wet Environments

Tsubasa Adachi, Koki Ozawa, Shoma Kamanoi, Junya Yoshida, Riku Sasaki, Yasuyuki Miura, Yoshihito Takabe, Fabrice Domingues Dos Santos, Huang Tong, Atsushi Miyabo, Yasunori Takeda, Hiroyuki Matsui, and Tomohito Sekine*

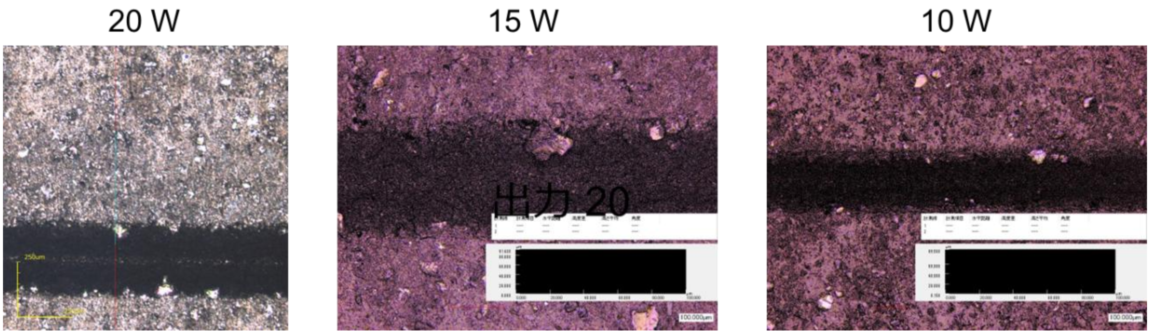

Figure S1. Relationship between laser power and pattern cleanliness. As laser power increases, eroding material in the depth direction becomes easier for the laser, resulting in fewer rubber remnants around the groove. This enhancement also improves the linearity of the pattern. However, excessively high output power can diminish linearity; thus, an optimal range of 20–30 W was identified for this study.

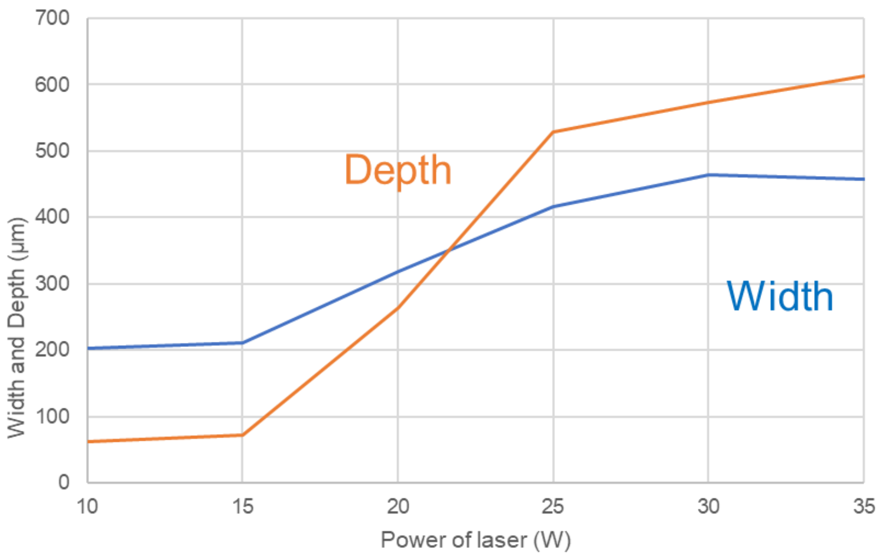

Figure S2. Relationship between groove depth and width of the fingerprint pattern and laser power. The output power of the laser cutter utilized in this study correlates with the groove depth and width of the pattern. A groove width of approximately 400 µm and a depth of approximately 500 µm were selected, as these dimensions produced the cleanest pattern.

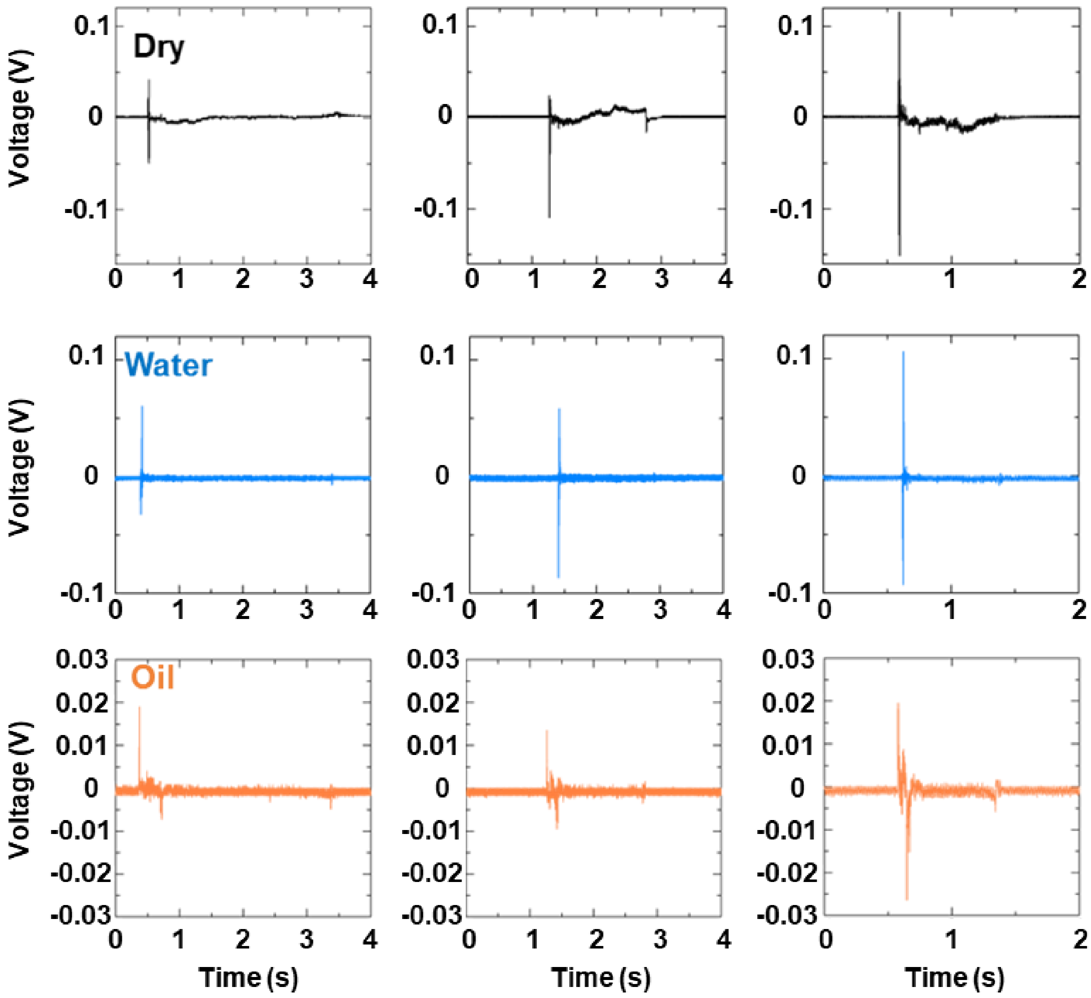

Figure S3. Signal intensity versus scanning phase. In this study, the scanning speed of the artificial finger was set at 50 mm/s. When the scanning speed was altered to 25 and 100 mm/s, the amplitude and frequency of the signal remained largely unchanged, but the amplitude slightly increased at higher scanning speeds. However, a speed of 50 mm/s is considered optimal as higher speeds may introduce noise. Spikes observed at the beginning of each scan are attributable to the piezoelectric properties of the sensor and do not affect the amplitude oscillation signals during scanning.

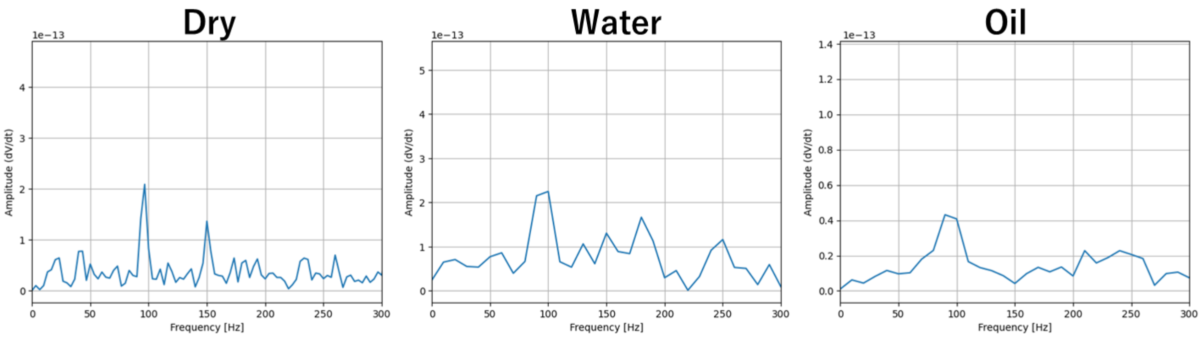

Figure S4 Results of the FFT applied to the slip signal shown in Figure 3d. The FFT was performed on the differentiated slip signal, and the analysis was conducted over an arbitrary 0.3 s segment within the slip portion of each dataset. Across all conditions, the frequency exhibiting the highest amplitude was approximately 100 Hz.

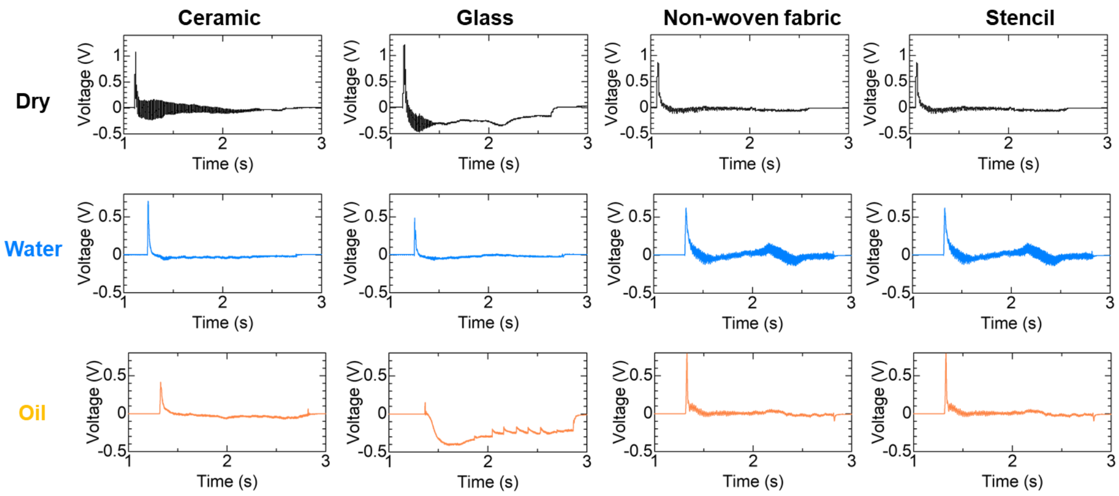

Figure S5. Signals used for AI in Fig. 4. In this analysis, 10 signals were acquired from dry, water-wetted, and oil-wetted surfaces, including the signals presented in Fig. 3. These signals were then utilized for machine learning. All signals were recorded under identical conditions, with no notable differences in waveforms observed within each set of 10 signals.

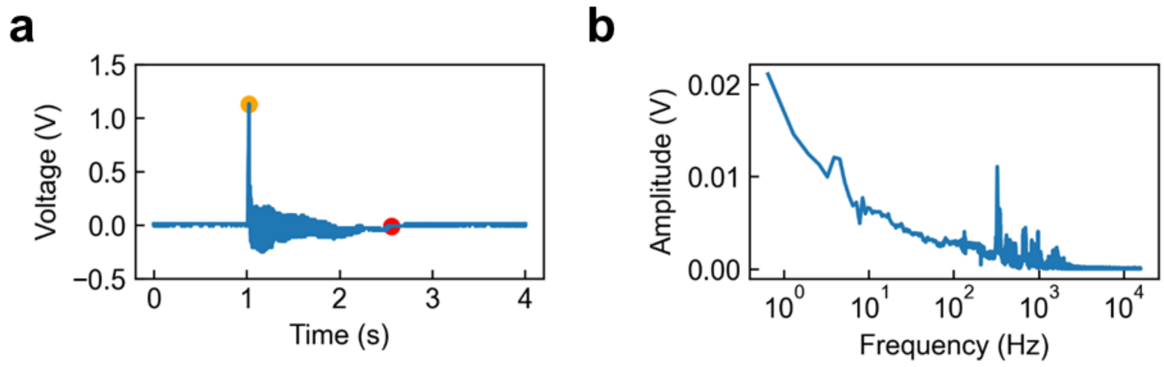

Figure S6. Example of the raw waveform of the sensor output during pressure application. (a) The starting point of pressure application was detected as the first peak (orange circle). 48,000 data points (~1.5 s) from the starting point were used for machine learning. The red circle indicates the last point. (b) Fourier transform of the waveform. The following features were used in machine learning: The mean, median, first quantile, third quantile, standard deviation, minimum, maximum, skewness, and kurtosis of raw waveform and its Fourier transform.

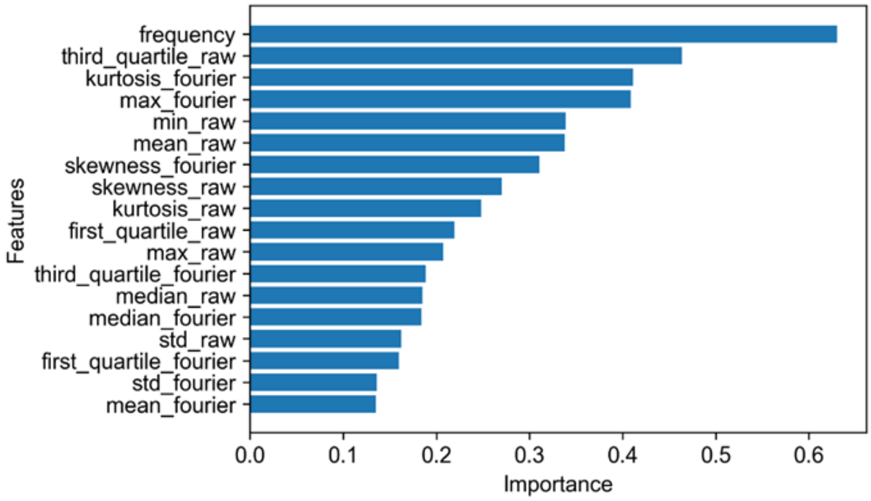

Figure S7. Impurity-based feature importance analysis for the machine learning model.
